# Crotoxin B from the South American Rattlesnake *Crotalus vegrandis* Blocks Voltage-Gated Calcium Channels Independent of Its Intrinsic Catalytic Activity

**DOI:** 10.3390/toxins18010036

**Published:** 2026-01-10

**Authors:** Markus Eicheldinger, Erick Miranda-Laferte, Francisco Castilla, Nadine Jordan, Beatrix Santiago-Schübel, Patricia Hidalgo

**Affiliations:** 1 Institute of Biological Information Processing (IBI-1)—Molecular and Cellular Physiology, Forschungszentrum Jülich, 52425 Jülich, Germany; m.eicheldinger@fz-juelich.de (M.E.); e.miranda.laferte@fz-juelich.de (E.M.-L.); n.jordan@fz-juelich.de (N.J.); 2 Institute of Biological Information Processing (IBI-7)—Structural Biochemistry, Forschungszentrum Jülich, 52425 Jülich, Germany; b.santiago-schuebel@fz-juelich.de; 3 Institute of Biochemistry, Heinrich-Heine University, 40225 Düsseldorf, Germany

**Keywords:** snake venom, *Crotalus vegrandis*, crotoxin, basic phospholipase A2 subunit, Ca_V_ channels

## Abstract

Neurotoxicity following South American *Crotalus* rattlesnake bite is primarily caused by crotoxin, the most abundant component in their venom. Despite the central role of voltage-gated calcium channels (Ca_V_) in neurotransmission, direct targetability by crotoxin has been poorly explored. Crotoxin is a non-covalent heterodimer formed by an acidic subunit (CA) and a basic toxic phospholipase A_2_ subunit (CB). Here, we chromatographically isolated the CB subunit from *Crotalus vegrandis* and studied its effect on Ca_V_ heterologously expressed in tsA201 cells using the whole-cell patch-clamp technique. Mass spectrometry analysis identified a protein that matched with 97% sequence coverage the CBc isoform from *Crotalus durissus terrificus*. Isolated CB exhibited moderate phospholipase activity that was not correlated to its cytotoxic effect on cultured tsA201 cells. Using Ba^2+^ as a charge carrier to prevent the enzymatic activity, we found that CB inhibited currents mediated by the N-type Ca_V_2.2 and Ca_V_1.2 L-type calcium channels, in a dose–dependent manner, with higher potency for the latter, and negligible changes in the voltage dependence of channel activation. Our results reveal a novel phospholipase-independent biological activity and a molecular target of CB providing new insights into the pathophysiology of *Crotalus* snakebite envenoming with potential clinical therapeutic implications.

## 1. Introduction

Snakebites from South American rattlesnakes (genus *Crotalus*) are highly lethal through a variety of systemic effects including neurotoxicity, myotoxicity, and hemotoxicity [1,2,3]. Crotoxin is the most abundant proteic neurotoxin of *C. durissus* and *C. vegrandis* venom and the main cause for the high neurotoxic effect following crotalus envenoming [3,4].

Crotoxin is a complex composed of an acidic (CA), nontoxic and noncatalytic subunit, and a basic (CB), weakly toxic, phospholipase A_2_ (PLA2) active subunit [5,6]. CA promotes the interaction of CB with specific crotoxin receptors at the cell membrane, substantially potentiating the toxicity of the heterodimeric complex and leading to the dissociation of the subunits [3,7,8]. The PLA2 catalytic activity of CB is strictly dependent on the presence of Ca^2+^ ions and, in the absence of CA, the different CB isoforms display similar three-dimensional structure, enzymatic activity and toxicity but different biological effects [6,8,9,10,11,12,13]. Among the latter, uncomplexed CB exerts cardiotoxic, analgesic, nephrotoxic, immunomodulatory, anti-inflammatory, anticoagulant, and anti-tumour effects that may or may not be dependent on its PLA2 activity [5,6,9,14,15,16]. The fact that the variety of pharmacological actions of CB are not readily associated with the potency of its enzymatic activity suggests that some of the effects are determined by interactions with different partners at the cell surface of the target tissue [9,15,17]. Those interactions, either with lipids or proteins, are mediated by regions outside the catalytic site [12].

Isolated CB from *C. durissus terrificus* exhibits distinctive presynaptic actions in ex vivo preparations derived from the peripheral and central nervous system; it reduces evoked acetylcholine neurotransmitter release at the neuromuscular junction, whereas it stimulates glutamate release in rat cerebrocortical synaptosomes in a Ca^2+^- and PLA2 activity-dependent manner, though at ten times higher concentrations than CA/CB heterodimer [18,19]. Despite the main role of voltage-gated calcium channels (Ca_V_) in neurotransmission, excitation–contraction and excitation–secretion coupling in nerve, muscle and gland cells, respectively, a direct effect of isolated CB on Ca_V_ channels has not been demonstrated. Here, we applied electrophysiological methods to investigate the effect of CB purified from *C. vegrandis* on canonical presynaptic and postynaptic Ca_V_ channels.

Ca_V_ channels are heteromultimers composed of a main multispanning transmembrane Ca_V_α_1_-pore-forming subunit encompassing the voltage sensor and drug-binding sites, and several nonhomologous accessory subunits, Ca_V_β, Ca_V_α_2_δ and Ca_V_γ, that regulate the conduction properties and surface expression of the channel [20]. Ca_V_α_1_ and Ca_V_β constitute the functional core of the high-voltage activated calcium channels, which are grouped into two subfamilies; Ca_V_1.x or L-type encoded by Ca_V_1.1 to 1.4 pore-forming subunits and Ca_V_2.x encoded by Ca_V_2.1 (P/Q-type), Ca_V_2.2 (N-type), and Ca_V_2.3 (R-type) subunits [21,22]. In the central nervous system, Ca_V_2.2 channels are localized in the active zone at the presynaptic terminal and calcium entering through these channels directly triggers neurotransmitter release, whereas Ca_V_1.2 is mainly localized postsynaptically in dendritic spines [23,24]. In neurons, Ca_V_1.2 contributes to the excitation–transcription coupling and plays important roles in neuronal function, such as long-term synaptic plasticity and neurodevelopment [24,25]. Furthermore, the Ca_V_1.2 channel is the main Ca_V_ channel expressed in cardiac muscle cells, where it mediates excitation–contraction coupling [26].

*C. vegrandis*, commonly known as the Uracoan rattlesnake, is geographically limited to a semiarid savanna region near Uracoa in Venezuela [1]. This unique distribution and specific habitat make its venom attractive to uncover new compounds targeting Ca_V_ channels since venom composition varies with geographical distribution as well as with sex and age [27,28,29,30].

The purified CB from *C. vegrandis* displayed PLA2 activity and cytotoxicity and, most notably, isolated CB directly blocked Ca_V_1.2 and Ca_V_2.2 channels heterologously expressed in tsA201 cells. Blockade was not associated with the intrinsic PLA2 catalytic activity.

Our results expand the current understanding of the pharmacological and biological impact of crotoxin and may improve the clinical management and outcome of *C. vegrandis* snakebite envenoming events.

## 2. Results

### 2.1. Isolation of Highly Purified CB Subunit of Crotoxin from C. vegrandis Whole Venom Extract

**Figure 1:**
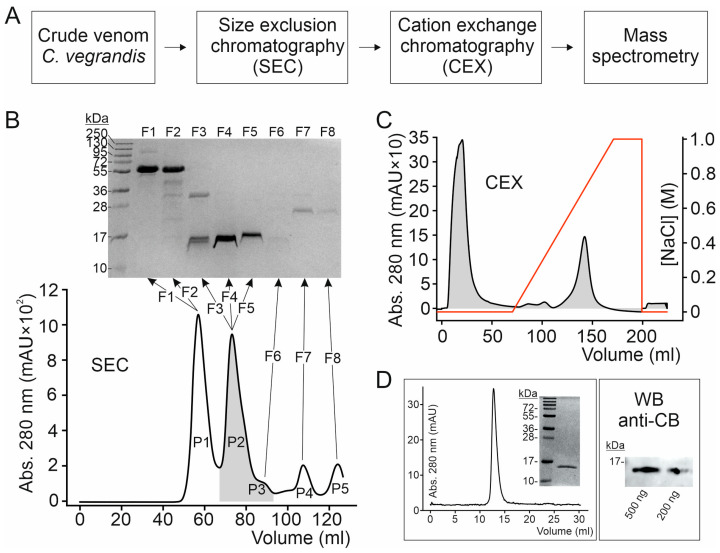
Purification of the basic subunit (CB) of CrTx from *C. vegrandis* crude venom extract. (**A**) Scheme of the strategy used to purify CB from the *C. vegrandis* crude venom extract obtained. The crude extract of *C. vegrandis* venom (obtained from Latoxan^TM^) was reconstituted in 100 mM ammonium acetate, pH 6.8, and subjected to size-exclusion chromatography (SEC) followed by cation exchange chromatography (CEX) and mass spectrometry analysis of the purified protein. (**B**) Elution profile from SEC (120 mL bed-volume Superdex 75 16/60) of the whole venom extract with the corresponding reducing SDS-PAGE gels of the indicated fractions. (**C**) Elution profile from CEX column of fractions underlying P2 (labelled in grey in panel B). CEX elution was performed with a linear NaCl gradient (red line in the CEX chromatogram). (**D**) Analytical SEC column (24 mL bed-volume Superdex 75 10/30) elution profile of the purified CB subunit with the corresponding reducing SDS-PAGE gel and the Western blot (WB) analysis detected with an antibody anti-CB subunit of crotoxin from *C. durissus terrificus*. In all gels, the numbers denote the size of molecular weight standards (MW).

To purify CB, we used the commercially available venom extract from *C. vegrandis* available from Latoxan^TM^. The crude extract was fractionated via size exclusion chromatography (SEC) onto a 120 mL bed-volume Superdex 75 16/60 column, followed by cation exchange chromatography (CEX), considering the positively charged nature of CB. The eluted fractions were analyzed by SDS-PAGE and the candidate protein was subjected to mass spectrometry analysis (Figure 1A).

Two major well-resolved chromatographic peaks, namely P1 and P2, eluted from the SEC column loaded with the whole venom extract (Figure 1B). Fractions under these peaks were collected and resolved in reducing SDS-PAGE. The proteins underlying P2 migrate below the 17 kDa molecular mass marker in SDS-PAGE gel and, therefore, are comparable to the molecular mass of the putative CB subunit of a crotoxin homologue from the venom of *C. vegrandis* [4]. The P2–containing fractions were collected, pooled and loaded onto a CEX column. Bound proteins were eluted with a linear NaCl gradient, resulting in one main peak eluting at 700 mM NaCl concentration that contained a highly purified protein (at about 15 kDa) as assessed by reducing SDS-PAGE (Figure 1B). The purified protein eluted as a single monodisperse peak in analytical SEC, indicating a homogeneous protein with no indication of protein aggregation, and when analyzed by Western-blot, it was detected by the anti-CB antibody against the CB subunit from *C. durissus terrificus* (Figure 1B).

**Figure 2:**
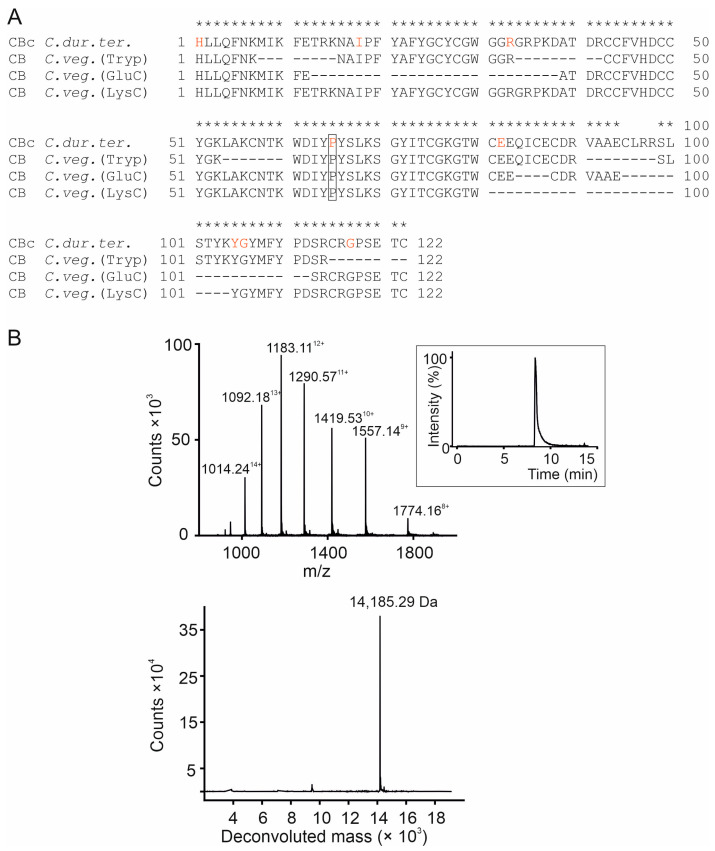
Mass spectrometry analysis of crotoxin CB subunit from *C. vegrandis*. (**A**) Alignment of the amino acid sequence of the crotoxin CB subunit from *C. durissus terrificus* (*C. dur. ter.*) (UniProt accession number P62022) and the cleavage peptides derived from the CB subunit from *C. vegrandis* (CB *C. veg.*) identified by mass spectrometry after digestion with Trypsin (Tryp), GluC, and LysC proteases. The asterisks denote identity between the amino acids of the peptide fragments identified by mass spectrometry after cleavage of CB *C. veg.* and CB *C. dur. ter.* sequence. The four consecutive amino acids (95–98) were not identified by the MaxQuant software (version 2.4.2.0). The dashes correspond to amino acids that were not identified after cleavage with the indicated protease. The eight variable residues among the four natural CB isoforms are labelled in red with the CBc-specific proline enclosed in a rectangle. (**B**) Mass determination of CB *C. veg* showing its ESI-MS spectra (upper panel) and deconvoluted mass spectrum (bottom panel). The chromatography profile of CB *C. veg.* on C8 reversed-phase chromatography is shown in the inset.

Mass spectrometry analysis of CB purified from *C. vegrandis* digested with Trypsin, GluC and LysC proteases identified peptide fragments that matched 97% of the amino acid sequence of CBc subunit from *C. durissus terrificus* (UniProtKB P62022) (Figure 2A). Moreover, the average molecular mass of the toxin was determined to be 14,185.29 Da (Figure 2B), which precisely matches the predicted average molecular mass of 14,185.35 Da of CBc from *C. durissus terrificus*.

The four different natural CB isoforms, CBa_2_, CBb, CBc, and CBd, differ from each other in eight amino acids spanning along the 122 residues of the CB from *C. durissus terrificus* [6,12] (Figure 2A). Mass spectrometry analysis confidently identified the eight variable amino acids that include the proline residue at position 65 that is only found in the CBc isoform (Figure 2A).

Altogether, these results confirm the occurrence of CB in the venom extract from *C. vegrandis*, as previously reported [1,4,31], and demonstrate the presence of CBc-like isoform.

### 2.2. The Protein Composition of the Crude Venom Does Not Vary with the Sex and Age of C. vegrandis

**Figure 3:**
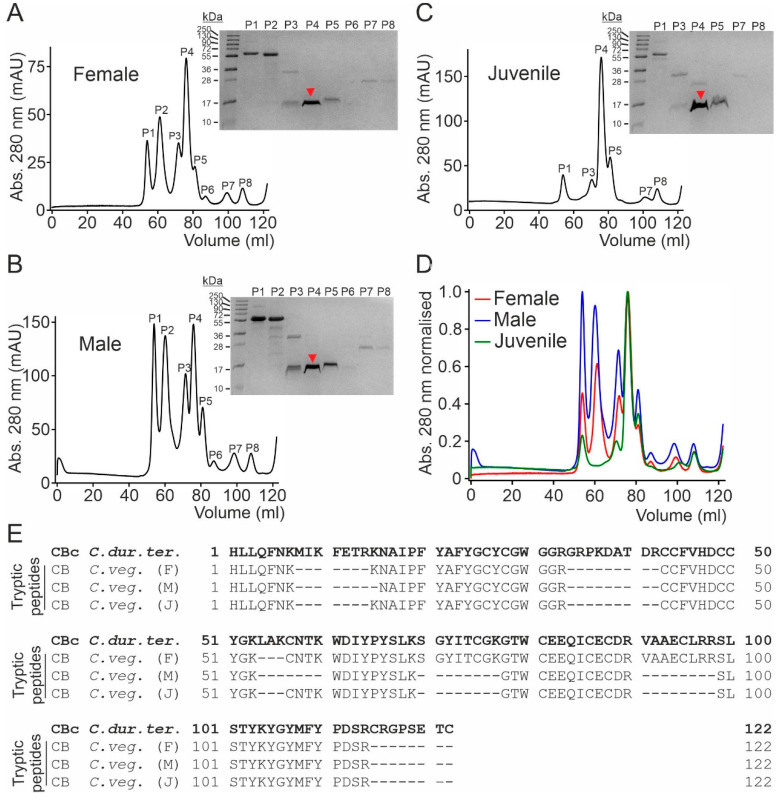
Comparison of the crude venom composition from female, male and juvenile *C. vegrandis*. (**A**) SEC elution profile of the crude venom sample collected from a female specimen and SDS-PAGE gel of the indicated peak fractions eluted from the Superdex-75 column (120 mL bed volume). (**B**) SEC elution profile and SDS-PAGE gel of the indicated peak fractions from the crude venom sample collected from a male specimen. (**C**) SEC elution profile and SDS-PAGE gel of the indicated peak fractions from the crude venom sample collected from juvenile specimens. (**D**) Overlay of the SEC chromatograms of the three indicated samples normalized to the maximum peak amplitude. The protein band migrating at ~15 kDa contained in peak 4 in all samples (denoted with a red arrow in each gel) was excised and subjected to mass spectrometry analysis. In all gels, the numbers denote the size of molecular weight standards (MW). (**E**) Alignment of the amino acid sequence of the crotoxin CB subunit from *C. durissus terrificus* (*C. dur. ter.*) (UniProt accession number P62022) and the tryptic peptides obtained from the mass spectrometry analysis from the female, male and juvenile samples from *C. vegrandis* (*C. veg.*).

To evaluate potential variations related to sex or age, we assessed the constituents of crude venom extracts collected from male, female and juvenile specimens of *C. vegrandis* (Gifftierhaus Eimsheim e.V). The three samples were separately fractionated via SEC, and the eluted fractions were analyzed by SDS-PAGE, followed by mass spectrometry (Figure 3). The elution profiles of the female, male and juvenile samples showed several chromatographic peaks with virtually identical retention volumes among the three samples and protein migration pattern in SDS-PAGE (Figure 3A–C). The overlap of the elution profiles shows that they mainly differ in their relative abundance, with the most prominent peak, particularly in the female and juvenile samples, eluting at around 78 mL and migrating at about 15 kDa in reducing SDS-PAGE (Figure 3D). From all three gels, the major band at around 15 kDa was excised, subjected to tryptic digestion and mass spectrometry analysis. This analysis resulted in the identification of several tryptic peptides that highly matched the amino acid sequence of the CBc subunit from *C. durissus terrificus* (Figure 3E).

### 2.3. Phospholipase Activity and Cytotoxicity of CB Subunit Purified from C. vegrandis

**Figure 4:**
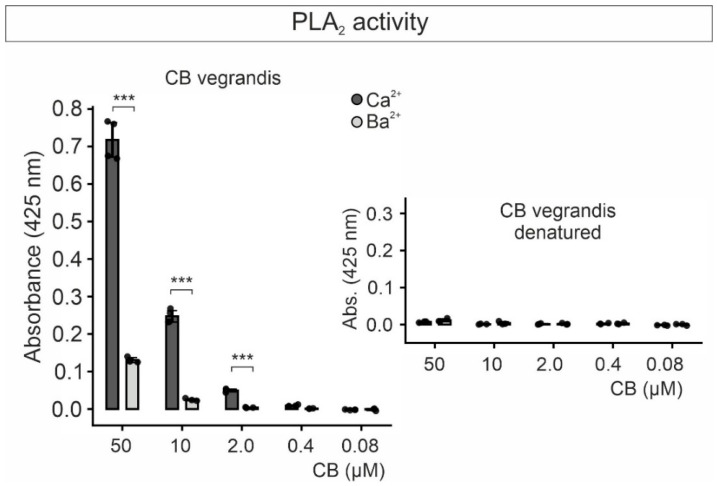
Purified CB from *C. vegrandis* exhibits Ca^2+^-dependent phospholipase A_2_ (PLA2) enzymatic activity. Graph of the PLA2 activity colorimetrically measured at 425 nm at the indicated CB concentrations before (**left**) and after heat denaturation (**right**), and either in the presence of Ca^2+^ or Ba^2+^. Each data point in the graph represents the average absorbance value at 425 nm from one experiment with three replicates. Each experiment was repeated three times. Statistical comparison between samples was performed using Welch’s *t*-test; *** (*p* < 0.001).

The PLA2 capability of the purified CB from *C. vegrandis*, at concentrations between 0.08 µM and 50 µM, was tested using a colorimetric assay [32,33]. In the presence of Ca^2+^ (10 mM), CB displayed a relatively robust PLA2 activity at the highest concentration tested that decreased considerably with decreasing CB concentrations up to undetectable levels at concentrations lower than 0.4 µM (Figure 4). When Ca^2+^ was substituted in the assay solution with an equimolar concentration of Ba^2+^ to inhibit PLA2 action [13] the enzymatic activity was indeed dramatically reduced at all CB concentrations (Figure 4). At 10 µM CB PLA2 activity was barely detectable and about ten-fold lower as compared with the one estimated in the Ca^2+^-containing solution. Heat denaturation of CB completely abolished the enzymatic activity in both, Ca^2+^- and Ba^2+^-containing solutions, indicating that lipolysis was indeed mediated by the protein (Figure 4). Therefore, CB purified from *C. vegrandis* retained its catalytic action, and consequently its overall three-dimensional architecture.

**Figure 5:**
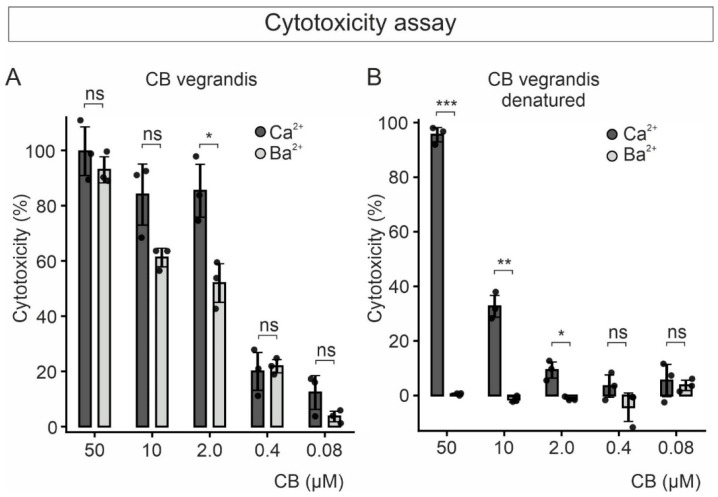
Toxicity of purified CB to tsA201 cells bathed in extracellular solution containing either Ca^2+^ or Ba^2+^. (**A**) Graph of the cytotoxic effect, measured colorimetrically by means of lactate dehydrogenase (LDH) activity, versus concentration of purified CB after 90 min incubation of the cells in either Ca^2+^- or Ba^2+^-containing extracellular solution. (**B**) Cytotoxicity of heat-denatured CB, at the indicated concentrations, to cells bathed in Ca^2+^- or Ba^2+^-containing extracellular solution. Each dot in the graph corresponds to the average percent cytotoxicity (see Materials and Methods) from one experiment with three replicates. Each experiment was repeated three times. Statistical comparison between samples was performed using Welch’s *t*-test; * (*p* < 0.05), ** (*p* < 0.01), *** (*p* < 0.001) and ns, not significant.

We next examined the cytotoxicity induced by the CB from *C. vegrandis* using a commercially available colorimetric kit (LDH-Cytox^TM^) that measures the lactate dehydrogenase (LDH) activity resulting from the release of the enzyme from damaged cells. In contrast to the PLA2 activity, CB exhibited rather comparable cytotoxic activity in solutions containing 10 mM of either Ba^2+^ or Ca^2+^ (Figure 5A). Whereas the PLA2 activity of CB is rather insignificant in the presence of Ba^2+^, its cytotoxic effect still takes place to a considerable extent, suggesting the occurrence of non-enzymatic mechanisms contributing to cytotoxicity.

Heat denaturation of CB—at all concentrations tested—blunted cell death in Ba^2+^- but not in Ca^2+^-containing solution (Figure 5B). Concentrations of the denatured toxin higher than 2.0 µM were able to exert cytotoxic effects. It is likely that high concentrations of Ca^2+^ might increase the thermal stability of the protein or induce toxic conformational changes.

### 2.4. Internalization of CB Purified from C. vegrandis in HeLa Cells

It has been suggested that CB interacts with extracellular and intracellular biological targets and thus, it must be internalized into cells to exert some of the effects [18,34]. Internalization of CB sourced from *C. durissus terrificus* into cerebellar granule cells [18] and mammary epithelial cells [35] has been previously shown.

**Figure 6:**
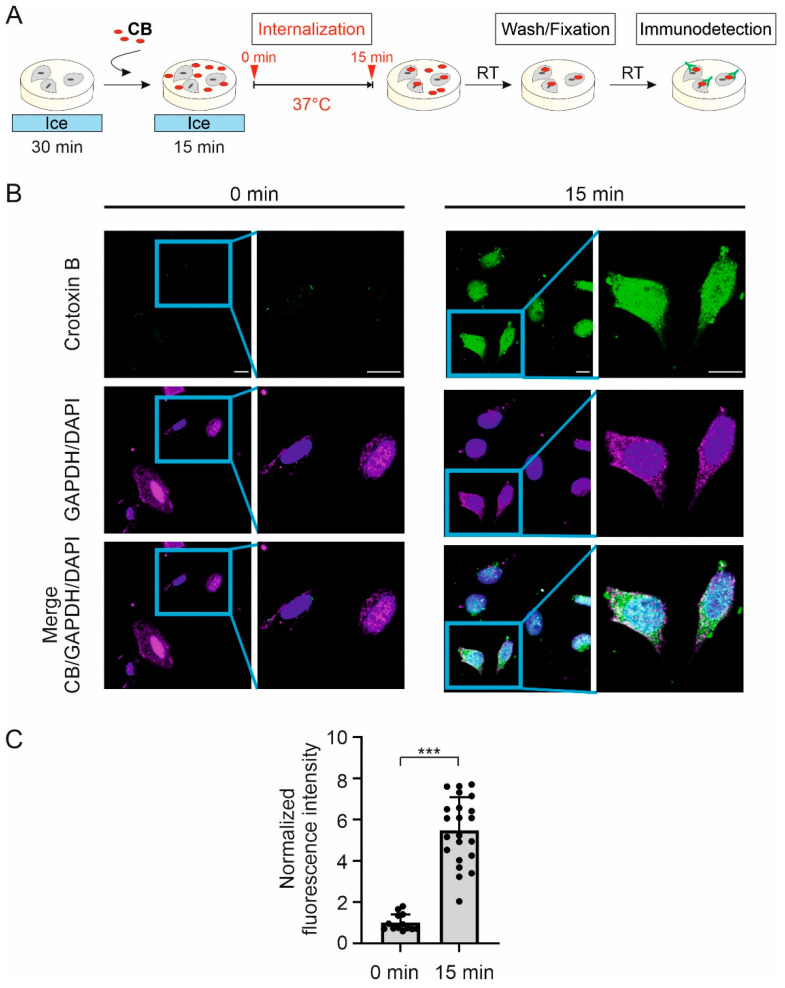
Crotoxin B purified from *C. vegrandis* is internalized in HeLa cells. (**A**) Schematic of the experimental design for crotoxin B (CB) internalization assay. Cells were incubated on ice for 30 min, exposed to CB and incubated on ice for an additional 15 min. Then cells were transferred to 37 °C for 15 min to allow CB internalization (t = 15 min). Time zero (t = 0 min) was taken before the transfer of cells to 37 °C. After several washes, cells were fixed and fluorescently stained for CB, intracellular GADPH and DAPI nuclei marker. (**B**) Laser scanning confocal images of representative HeLa cells stained for CB (green), GADPH (magenta), and DAPI (blue) at t = 0 and t = 15 min (after exposure to CB at 37 °C). Each image is shown with an enlarged view of the marked square region. Scale bar, 15 µm. (**C**) Graph of the CB intracellular fluorescence intensity normalized to the average intensity measured at t = 0. The number of cells measured at t = 0 and t = 15 min was 13 and 22, respectively. Statistical comparison between samples was performed using Welch’s *t*-test; *** (*p* < 0.001).

We here tested whether purified CB is internalized by HeLa cells. HeLa cells were chosen over tsA201cells because they exhibit a larger cytosol to nuclear ratio that allows better visualization of internalized proteins. Cells were incubated with 1µM CB on ice to inhibit endocytosis and transferred to 37 °C for 15 min to allow internalization of CB. Then cells were fixed and immunostained (Figure 6A). Control cells were fixed before the transfer to 37 °C (t = 0). The immunostained cells were visualized using laser-scanning confocal fluorescence microscopy.

Following the 15 min incubation at 37 °C with CB, the distribution of the fluorescent signal from CB overlaps with the signals of the intracellular GAPDH and the DAPI nuclear marker, indicating that CB was internalized into the cell and localized to the cytoplasm and nucleus (Figure 6B). In control cells, the fluorescence signal from CB was barely visible and significantly lower than that at 15 min (Figure 6B,C). This result demonstrates the capability of CB from *C. vegrandis* to be internalized, independently of CA.

### 2.5. Effect of CB Purified from C. vegrandis on Ca_V_2.2 and Ca_V_1.2 Calcium Channels Expressed in tsA201 Cells

Given that phospholipid hydrolysis leads to damage of the plasma membrane, precluding electrophysiological recordings, we measured ion currents through Ca_V_ channels using the whole-cell patch clamp technique with Ba^2+^ (instead of Ca^2+^) as charge carrier. Replacement of Ca^2+^ by Ba^2+^ successfully inhibited the PLA2 activity of CB (Figure 4). Currents were recorded from Ca_V_2.2 and Ca_V_1.2 channel complexes heterologously expressed in tsA201 cells after transfection with either Ca_V_2.2/Ca_V_β_4_ or Ca_V_1.2/Ca_V_β_2_ encoding plasmids. Ca_V_β subunit is included since it is required for plasma membrane targeting and normal function of the Ca_V_α_1_ pore-forming subunits, with Ca_V_β_4_ and Ca_V_β_2_ displaying preferences for Ca_V_2.2 and Ca_V_1.2, respectively [21,36].

**Figure 7:**
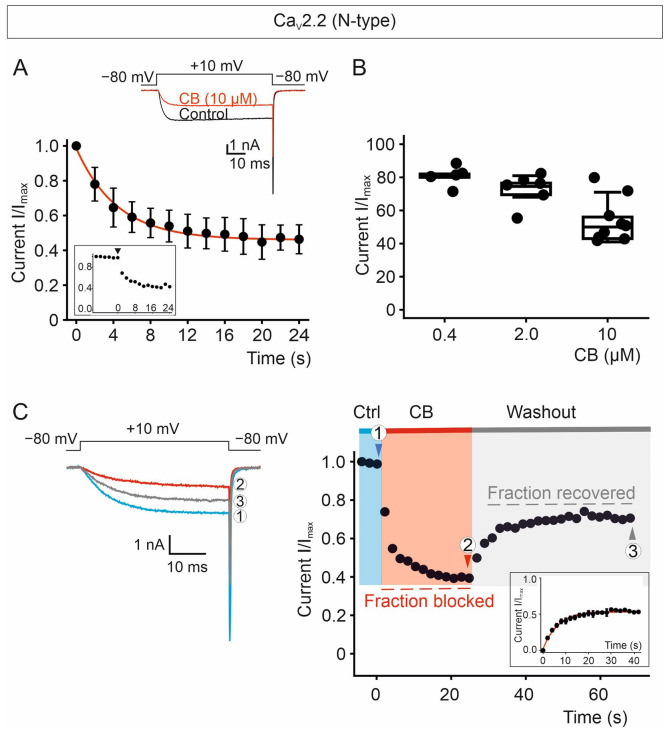
Time course of Ca_V_2.2 channel inhibition by CB purified from *C. vegrandis* and its recovery. (**A**) Average time course of inhibition of Ca_V_2.2-mediated currents by 10 µM of CB and representative ionic current traces elicited by the one voltage pulse, shown above the traces, to +10 mV from a holding potential of −80 mV repetitively applied every 2 s, in the absence (black trace) and presence (red trace) of CB. The inset shows the time course of a representative Ca_V_2.2-expressing cell perfused at time = 0 (denoted by an arrowhead) with 10 µM of CB. Data are presented as mean ± SEM; number of recorded cells = 14. (**B**) Concentration dependence of the steady-state Ca_V_2.2-current inhibition (mean ± SEM) by CB at 0.4, 2.0 and 10.0 µM; number of recorded cells = 5, 6 and 9, respectively. (**C**) Representative current traces and time course of Ca_V_2.2 channel block and its relief. CB blockade and its relief were monitored by recording the current response elicited by the one voltage step. The cell was subjected to continuous perfusion with control solution (Ctrl) containing 10 µM of CB (①). After achieving a steady-state blockade (②), the cell was moved to the control solution perfusion line to wash out the toxin (③). The inset shows the average time course of the relief from the block of Ca_V_2.2 channels. Data are presented as mean ± SEM; number of recorded cells = 5. Ba^2+^ was used as the charge carrier.

Whole-cell recordings from cells expressing the Ca_V_2.2 channel complex showed ionic currents evoked by a voltage step to +10 mV from a holding potential of −80 mV, which were significantly reduced upon continuous perfusion of the cell with 10 µM of CB (Figure 7A). To verify that steady-state blockade was achieved, the time course of CB-mediated current inhibition was monitored by recording the current responses elicited by the voltage step to +10 mV repetitively applied every 2 s. By perfusion with 10 µM of CB, a steady-state inhibition was reached after about 12 s, resulting in a current decrease of approximately 50% (Figure 7A). Lower concentrations of CB, namely 0.4 µM and 2.0 µM, inhibited currents to a lesser extent (Figure 7B). Due to cell damage, recordings at CB concentrations higher than 10 µM were shunned.

To study the recovery of the block, once a steady-state blockade was established using 10 µM CB, the toxin was washed out by perfusing the cell expressing Ca_V_2.2 channels with control solution (Figure 7C). Ca_V_2.2-mediated current inhibition was partially reversible upon removal of CB, with approximately 50% recovery. Recovery from the block occurs within seconds; this rapid reversal is inconsistent with the irreversible damage characteristic of cytotoxicity.

**Figure 8:**
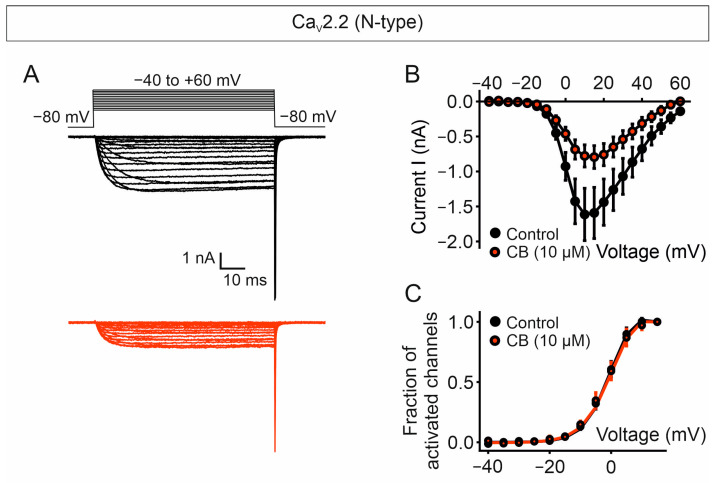
CB purified from *C. vegrandis* inhibits currents through Ca_V_2.2 N-type calcium channel without altering the voltage dependence of activation of the channel. (**A**) Representative ionic current traces elicited by the voltage pulse protocol shown above, consisting of voltages from −40 to +60 mV in 5 mV increments from a holding potential of −80 mV, obtained from tsA201 cells expressing Ca_V_2.2 channel before (black) and after (red) perfusion with 10 µM CB. (**B**) Plots of ionic current (I) versus voltage obtained from cells before (black data points) and after (red data points) exposure to 10 µM CB. (**C**) Plots of the fraction of activated channels versus voltage obtained from cells in panel (**B**). Ba^2+^ was used as the charge carrier. Data points represent mean ± SEM; number of recorded cells = 9.

The voltage-dependent ionic currents in response to different voltage steps, from −40 mV to +60 mV, from a holding potential of −80 mV, in the absence and presence of CB, are shown in Figure 8A. The ionic current versus voltage (I/V) plot shows that CB reduces ionic currents amplitude at all voltages and with an average decrease of about 50% in the maximal (peak) current obtained at +10 mV (Imax, −1.61 ± 0.37 nA and −0.78 ± 0.16 nA; *p* = 0.0078, paired *t*-test, for control cells and CB-treated cells, respectively) (Figure 8B). Furthermore, virtually identical curves for the fraction of activated channels versus voltage were obtained for control cells and cells exposed to CB, indicating that CB-mediated current reduction is not caused by alterations in the voltage dependence of activation of the channel (Figure 8C).

**Figure 9:**
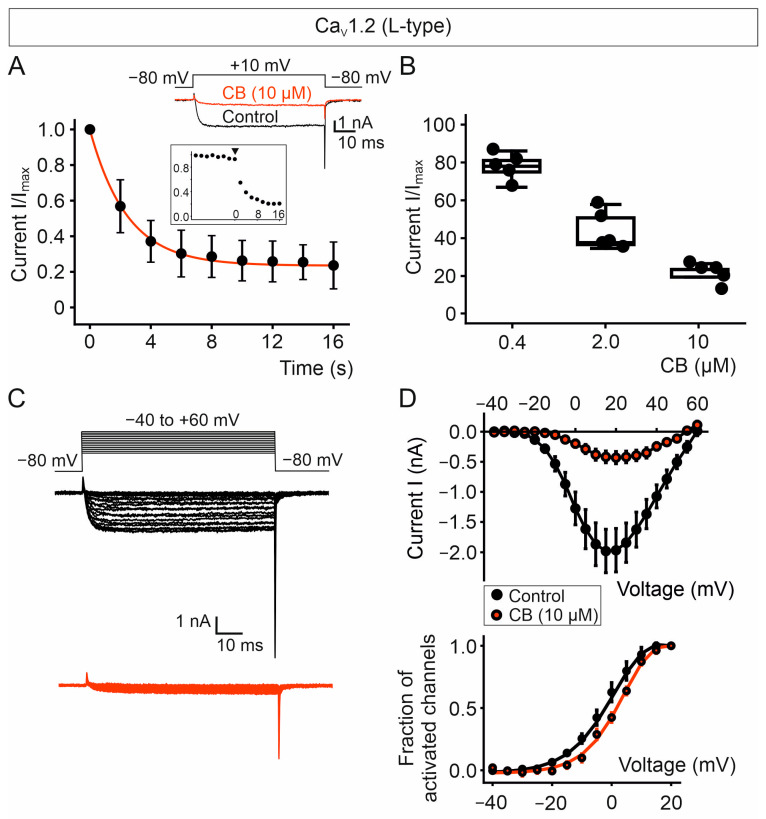
CB purified from *C. vegrandis* potently blocks Ca_V_1.2 L-type calcium channels. (**A**) Average time course of inhibition of Ca_V_1.2-mediated currents by 10 µM of CB and representative current traces elicited by the one voltage pulse (shown above the current traces) in the absence (black trace) and presence (red trace) of CB. The inset shows the time course of a representative cell expressing Ca_V_1.2 channel complexes. Perfusion with 10 µM of CB started at time = 0. Data are presented as mean ± SEM; number of recorded cells = 14. (**B**) Graph of the inhibition of Ca_V_1.2-mediated currents by 0.4, 2.0 and 10.0 µM of CB. Data are presented as mean ± SEM; number of recorded cells = 5 for each concentration. (**C**) Representative ionic current traces through Ca_V_1.2 channel complexes before (black data points) and after (red data points) perfusion with 10 µM CB. The voltage pulse protocol is shown above the current traces. (**D**) Plots of ionic current and fraction of activated channels versus voltage for cells either exposed (red data points) or not (black data points) to 10 µM of CB. Ba^2+^ was used as the charge carrier. Data are presented as mean ± SEM; number of recorded cells = 5.

As for the Ca_V_2.2 channel complex, we employed the single voltage protocol to monitor the temporal course of the current inhibition through Ca_V_1.2 complexes (Figure 9A). Blockade of Ca_V_1.2 currents after perfusion with 10 µM of CB reached an equilibrium within about 10 s with nearly 80% of current inhibition. CB inhibits the 50% of the Ca_V_1.2-mediated currents at a concentration of approximately 2.0 µM (Figure 9B), which is about five-fold less than the CB concentration needed to achieve the same current decrease in Ca_V_2.2-expressing cells. Therefore, CB appears to block Ca_V_1.2 with apparent higher potency than Ca_V_2.2 channels.

In the presence of 10 µM CB, the maximal current amplitude (measured at +15 mV) decreased 79% compared with control cells as shown in the ionic current versus voltage (I/V) plot for Ca_V_1.2 complexes (Imax, −1.98 ± 0.36 nA and −0.42 ± 0.10 nA; *p* = 0.0070, paired t-test, for control cells and CB-treated cells, respectively) (Figure 9C,D). The fraction of activated channels versus voltage plot obtained from CB-treated cells shows a modest rightward shift of 3.8 mV in the half-activation voltage compared to the control cells (Figure 9D). This relatively small change in the voltage dependence of activation in the presence of the CB subunit is not sufficient to account for the significant reduction in the current amplitude mediated by Ca_V_1.2 channels.

## 3. Discussion

The main finding of the present work is that the basic CB subunit of crotoxin from *C. vegrandis* suffices to block ion conduction through the voltage-gated Ca_V_1.2 and Ca_V_2.2 calcium channels in the tsA201 mammalian heterologous expression system. Ca_V_ block is independent of its intrinsic PLA2 enzymatic activity and the presence of the acidic CA subunit. To the best of our knowledge, this is the first study reporting direct inhibition of Ca_V_-mediated currents by an isolated CB subunit. The molecular mass of CB restricts its absorption into the bloodstream and its passage across the blood–brain barrier and entry into the central nervous system. It has been shown that a significant amount of the crotoxin is absorbed into the lymphatic system. Once in the bloodstream, its rapid clearance suggests it might move quickly towards its tissue targets [37,38]. The L-type calcium channels directly regulate the contraction of vascular smooth muscle, and their blockade will lead to muscle relaxation and vasodilation, which will further improve the movement of the toxin from the bloodstream into its specific tissue targets. On the other hand, although several studies have shown effects of crotoxin that suggest it reaches the central nervous system [39,40,41], whether or not CB actually crosses the barrier remains unresolved. If indeed CB has the ability to permeate the blood–brain barrier and access the central nervous system, then blocking of Ca_V_ channels would have a significant impact on the prey’s survival.

Blockade of Ca_V_2.2 occurs with no alterations in the voltage dependence of activation of the channel, compatible with the notion that CB operates as a pore blocker [42]. The incomplete recovery of Ca_V_2.2 channels from CB block suggests a state-dependent mechanism of block, which has been observed for peptide toxins that display preferential interaction with the inactivated state of the channel [43,44]. Future studies are required to determine potential CB-induced changes in the voltage dependence of channel inactivation, as such alterations would critically influence the amount of calcium entering the cell and, consequently, its availability for downstream signals. Despite the underlying mechanism, the blockade of Ca_V_2.2 channels anticipates an acute biological effect after exposure to the venom, given that CaV2.2 channels directly trigger neurotransmission; an incoming action potential into the nerve terminal opens CaV2.2 channels, allowing Ca^2+^ influx at the presynaptic nerve terminal, which in turn permits neurotransmitter release [23]. Therefore, inhibition of Ca_V_2.2 currents results in decreased calcium for neurotransmission with a concomitant impairment of synaptic activity.

The inhibition of Ca_V_2.2-mediated currents by CB demonstrated in this study is in apparent contrast to the increase in glutamate release induced by CB (from *C. durissus terrificus*) observed in rat cerebral cortex synaptosomes [18]. This effect depended on the PLA2 enzymatic activity of CB and on the entry of calcium via neuronal Ca_V_2.1 and Ca_V_2.2 channels. The authors proposed that the generation of arachidonic acid, facilitated by CB, stimulates Ca2+ permeation and, in turn, glutamate release [18]. A direct effect of CB on Ca_V_ channels was not explored. The study showed internalization of CB from *C. durissus terrificus* that is in line with our result showing that purified CB from *C. vegrandis* is internalized within minutes into HeLa cells. The capability of CB to enter into the cell and interact with intracellular targets [5,34,45] may expand not only the variety of biological actions but also confer the ability to induce diverse pharmacological effects at different time scales, i.e., fast inhibition of Ca_V_s from the extracellular side can be followed by intracellular actions acting directly or indirectly on a different target.

The current inhibition through Ca_V_1.2 is more potent than for Ca_V_2.2, and it is accompanied by a small right shift in the voltage activation curve, suggesting that changes in the gating of the channel might slightly contribute to current inhibition. An opposing effect on L-type calcium channels has been observed with the heterodimeric crotoxin (CA/CB subunits) from *C. durissus terrificus*, in which the toxin induced a two-fold increment in the peak current amplitude mediated by these channels, reported in neonatal rat cardiomyocytes [46]. Therefore, we attribute the different effects on Ca_V_-mediated currents to the presence of CA, which changes the *modus operandi* of CB, diversifying the crotoxin effects in different tissues [5,8].

In the heart, Ca_V_1.2 localizes to the transverse tubules of the cardiomyocytes, and its activation is mandatory for initiating cardiac contractions [26]. The upregulation of Ca_V_1.2 channels by β-adrenergic stimulation increases heart rate and contractile force, being central to the fight-or-flight response [47,48,49]. This response is activated upon perceived danger and is key for the survival of the animal during immediate stress, i.e., by a prey exposed to the predator. A blockade of the cardiac Ca_V_1.2 would profoundly interfere with this acute stress reaction. Additionally, it is well established that adrenaline strongly potentiates Ca_V_1.2 function during sympathetic stimulation; whether and how CB could alter the adrenergic enhancement of Ca^2+^ influx remains to be elucidated. It is worth mentioning that calmodulin, which is a key regulator of L-type channels gating, has been reported to be one of the intracellular acceptors for ammodytoxin, a presynaptic PLA2 neurotoxin from *Vipera ammodytes* [50,51]. In the future, it will be interesting to investigate a potential interaction of CB from *C. vegrandis* with calmodulin and its role in Ca_V_ modulation.

In the brain, Ca_V_1.2 sits at the postsynaptic side and does not contribute to neurotransmitter release but to calcium signalling, including gene transcription [24,25]. Additionally, isolated CB is mainly described as acting presynaptically, with postsynaptic effects [52] and binding to other ion channels in either heterologous expression or ex vivo systems have been reported [53,54]. A blockade of postsynaptic Ca_V_1.2 by CB may have a relatively long-term effect by influencing excitation–transcription coupling. Within this framework, it has been observed that two hours after intraperitoneal administration of crotoxin (CA/CB), rats develop behavioural alterations [39]. Moreover, these changes could be attributed to other effects of CB, a blockade of Ca_V_1.2 channels with a well-established role in stress response and brain function, which can explain the behavioural changes after *C. vegrandis* snakebite [36,55,56,57].

The PLA2 activity of CB in Ca^2+^-containing solution was relatively high at the highest concentration of toxin (50 µM) as compared with lower concentrations (≤10 µM). In Ba^2+^-containing solution, the PLA2 activity measured was negligible except at 50 µM CB. Heat-denatured CB exhibits no PLA2 activity in either Ca^2+^ or Ba^2+^ solution, demonstrating that the catalytic activity is solely mediated by the toxin. Despite the insignificant degree of catalytic activity of CB in Ba^2+^, its cytotoxicity levels were comparable to those obtained in Ca^2+^-containing solution, indicating that cytotoxicity is not caused primarily by its lipolytic activity. Results from several studies have suggested that CB can exert pharmacological effects in a phospholipase-independent manner by interacting with phospholipids even in the absence of divalent cations. Both binding to synaptic membranes and neurotoxicity, but not its phospholipase activity, are virtually lost upon tyrosine modification [13,58]. The most relevant tyrosine residue determining neurotoxicity and membrane binding affinity, at position 21, is present in the purified CB from *C. vegrandis* as assessed by mass spectrometry-analyzed tryptic peptides [58]. Therefore, we assume that, under our conditions, cytotoxicity of CB is not inherent to its catalytic activity but associated with its ability to interact and damage cell membranes [3,5,7,13,15,59,60]. Alternatively, we cannot fully discard that a minimal PLA2 activity may be sufficient for an early membrane perturbation effect promoting, among other actions, toxin internalization and a chain of intracellular events leading to further cell damage.

## 4. Conclusions

**Figure 10:**
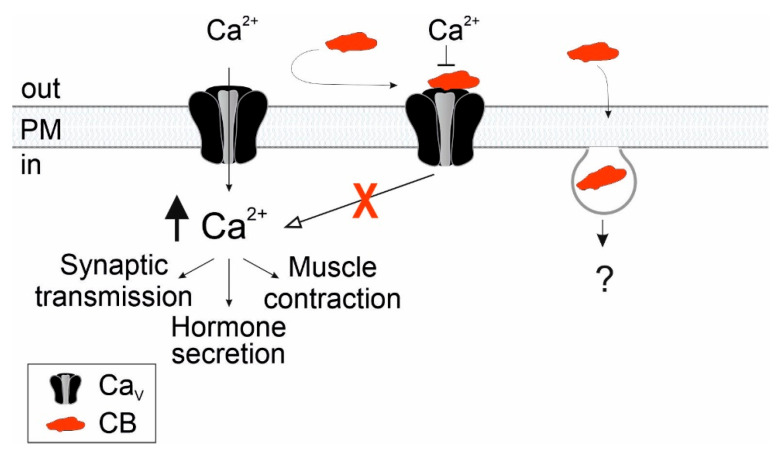
Model for the action and biological outcome of CB purified from *C. vegrandis*. CB from *C. vegrandis* (represented in red) interacts directly with Ca_V_ channels assembled at the plasma membrane (PM). Calcium entry through Ca_V_ calcium channels result in a transient increase in the intracellular Ca^2+^ concentration (black arrow) that initiates several cellular events such as synaptic transmission, hormone secretion and muscle contraction. CB blocks Ca_V_ channels, preventing calcium influx into the cell and inhibiting Ca^2+^-signalling processes mediated by the channel (red X). The mechanism of CB internalization and interactions with intracellular targets are not yet fully understood.

The present study reveals that Ca_V_ channels, which are intimately involved in neurotransmission and cardiac contraction, are molecular targets of the PLA2 subunit of crotoxin purified from *C. vegrandis*. The capability of CB to be internalized opens potential mechanisms of action; however, the fate of internalized CB and interactions with intracellular targets remain to be elucidated (Figure 10).

Our findings enlarge the list of ion channels targeted by the CB subunit of crotoxin and its pharmacological effects, providing new insights into the clinical aspects of rattlesnake envenomation.

## 5. Materials and Methods

### 5.1. Crotalus vegrandis Crude Venom Source

Two sources of venom were used: (a) venoms extracted from captive male, a female and five unsexed juveniles (13 weeks old) individuals of *C. vegrandis* [61] by Michael Steige from Gifttierhaus Eimsheim e.V. in Eimsheim (Germany). The taxonomy of the species follows the current status according to the Reptile Database (www.reptile-database.org, accessed on 14 October 2025). The venom from the three different species (male, female, and juvenile) was handled separately at all times and individually purified. After lyophilization using a Savant Speed Vac Plus SC110 A (Thermo Fisher Scientific, Dreieich, Germany), the crude venoms were stored at −80 °C until use. (b) Lyophilized venom from *C. vegrandis* was purchased from Latoxan Laboratory (Portes-lès-Valence, France). This venom was extracted from several specimens and corresponded to a mix of adult male and female. Upon arrival, it was stored at −80 °C until use.

### 5.2. Purification of Crotoxin CB Subunit from Crude Venom from Latoxan^TM^

The crude venom from Latoxan^TM^ was directly purchased from Latoxan Laboratory (Portes-lès-Valence, France). CB was purified from the crude venom using size exclusion chromatography (SEC) with a Superdex 75 16/160 GL column, 120 mL bed volume (GE Healthcare), followed by cation exchange chromatography (CEX) using a HiTrap SP Sepharose Fast Flow column (Thermo Fisher Scientific, Dreieich, Germany). The lyophilized venom was resuspended in 100 mM ammonium acetate buffer, pH 6.8, and loaded onto the Superdex 75 pre-equilibrated with the same buffer. The fractions containing CB, as judged by SDS-PAGE analysis, were pooled and loaded onto a CEX column equilibrated with 100 mM ammonium acetate buffer, pH 6.8. After washing with 100 mM ammonium acetate buffer, pH 6.8, the protein was eluted with a linear gradient from 0 to 100% with start and end buffer containing 100 mM ammonium acetate buffer, pH 6.8, not supplemented or supplemented with 1 M NaCl, respectively. The fractions containing CB, as judged by SDS-PAGE, were pooled, dialyzed overnight against ammonium acetate buffer without salt using a dialysis cassette (Slide-A-Lyzer 3.500 MWCO, Thermo Fisher Scientific, Dreieich, Germany) and concentrated using Amicon with 5.000 MWCO (Merck, Darmstadt, Germany). The concentrated CB sample was lyophilized overnight and stored at −80 °C until use.

### 5.3. Western-Blot Analysis of CB Purified from C. vegrandis

Purified CB was resolved in a 15% reduced SDS-PAGE and blotted onto a nitrocellulose membrane (GE Healthcare, Life Science, Solingen, Germany) for 1 h in transfer buffer (10 mM NaHCO_3_, 3 mM NaCO_3_, pH 9.9). Membranes were blocked in 5% BSA blocking buffer (10 mM Tris, 150 mM NaCl, 0.1% Triton X-100, pH 7.5) and incubated with anti-CB antibody against the CB subunit from *C. durissus terrificus* (Anti-South American/Mojave rattlesnake crotoxin antibody A10G, Hölzel, Germany) [62]. After several washes, membranes were incubated with anti-mouse IgG HRP-conjugated secondary antibody (Thermo Fisher Scientific, Dreieich, Germany). Protein bands were detected using a chemiluminescent detection kit (ECL Chemiluminescent Substrate, Thermo Fisher Scientific, Dreieich, Germany).

### 5.4. Phospholipase Activity of CB Purified from C. vegrandis

The phospholipase A_2_ enzymatic activity of the CB subunit of crotoxin purified from *C. vegrandis* crude venom obtained from Latoxan^TM^ (Portes-lès-Valence, France) was measured colorimetrically, as previously described [32,33]. The compound 4N3OBA (Merck, Darmstadt, Germany), used as substrate, was dissolved at a concentration of 50 mg/mL in chloroform. Aliquots of 80 µL (4 mg 4N3OBA) were evaporated under vacuum to remove chloroform and stored at −20 °C until use. The substrate was resuspended in 1 mL of assay buffer (10 mM Tris/HCl, 10 mM CaCl_2_ or BaCl_2_, 100 mM NaCl, pH 7.8), vortexed, centrifuged (2000× *g*, 2 min), and the supernatant was separated and used as a cleared substrate solution. The different concentrations of CB used for the assay were prepared in 10 µL aliquots and added to 190 µL of substrate solution placed in a 96-well plate, incubated for 40 min at 37 °C, and the absorbance at 425 nm was measured in a Tecan infinite^TM^ M1000 Pro plate reader. The concentration of purified CB was calculated using a molecular mass of 14.2 kDa and an extinction coefficient of 32,190 cm^−1^ M^−1^ [63,64].

### 5.5. Cytotoxicity Assay of CB Purified from C. vegrandis

The colorimetric cytotoxicity LDH-Cytox^TM^ assay (BioLegend, San Diego, CA, USA) was used to evaluate the cytotoxicity of the purified CB subunit of crotoxin from *C. vegrandis* crude venom (Latoxan^TM^, Portes-lès-Valence, France). Cytotoxicity was determined following the manufacturer’s instructions. Briefly, tsA201 cells were seeded in 10 cm dishes and grown in Dulbecco’s modified Eagle medium (DMEM, see below). The day of the assay, cells were harvested and resuspended in external solution (ES) used for whole cell recording containing 145 mM TEA-Cl, 10 mM BaCl_2_, 1 mM MgCl_2_, 10 mM HEPES, adjusted to pH 7.4 with TEA-OH, and transferred to a 96-well plate (12,500 cells per well). CB was added at different concentrations to the cell suspension and incubated for 90 min at 37 °C. After incubation, cytotoxicity of CB was determined by the absorbance measured at 490 nm as described in the cytotoxicity LDH-CytoxTM assay instruction manual in a Tecan infinite^TM^ M1000 Pro plate reader. The percentage of cytotoxicity is reported as the fraction between lysed cells by CB (CB) and a lysis buffer (B), with untreated cells (U) being subtracted from the corresponding absorbance values, as follows:

The average absorbance was calculated from three experiments with three replicates each.

$$Cytotoxicity\%=\frac{CB-U}{B-U}\times 100$$

### 5.6. cDNA Constructs

The following constructs were used to transiently transfect tsA201 cells for whole-cell patch-clamp recordings. Ca_V_2.2 (accession number: Q00975-1) fused to EGFP, Ca_V_1.2 (accession number: P15381) fused to mNeonGreen, Ca_V_β4 (accession number: O00305-2) fused to mCherry and Ca_V_β2a (Ca_V_β2-N3, accession number: Q8VGC3-2) fused to mRFP. Fusion of the channel subunits to fluorescence proteins facilitates recognition of transfected cells.

### 5.7. Cell Culture and Transfection

tsA201 cells (Merck, Darmstadt, Germany) were grown at 37 °C with 5% CO_2_ in Dulbecco’s modified Eagle medium (DMEM) supplemented with 10% fetal bovine serum (Merck, Darmstadt, Germany) and penicillin/streptomycin. For patch-clamp recordings, tsA201 cells were transiently co-transfected with either Ca_V_2.2-EGFP and Ca_V_β4-mCherry or Ca_V_1.2-mNeonGreen and Ca_V_β2a-mRFP. Cells were seeded onto 50 mm dishes and transfected using the calcium phosphate method. Cells were split 24 h after transfection and incubated for 24 h more before recordings. For laser scanning confocal fluorescence microscopy, cells were seeded in poly-lysine-coated 18 mm glass coverslips 24 h before use.

### 5.8. Internalization Assay and Laser Scanning Confocal Fluorescence Microscopy

Internalization assay was performed on HeLa seeded onto 1.8 mm coverslips. Cells were incubated in cold Hank’s Balanced Salt Solution (HBSS) on ice for 30 min to inhibit endocytosis. Then the cell bathing solution was replaced by cold HBSS supplemented with 1 µM of purified CB, and the cells were incubated on ice for an additional 15 min to allow CB binding to the cell surface. Then, the cells were transferred to 37 °C to allow internalization of CB. All the following steps were performed at room temperature. After three washes with PBS, cells were fixed with 4% paraformaldehyde (Merck, Darmstadt, Germany) in PBS for 10 min and washed several times. Coverslips that were fixed directly after ice incubation with CB were taken as a control (time = 0). After fixation, cells were permeabilized with 0.5% Triton X-100 in PBS, blocked using a filtered 5% solution in PBS of normal goat serum, NGS (Merck, Darmstadt, Germany), and incubated for 60 min with anti-crotoxin antibody (Anti-South American/Mojave rattlesnake crotoxin antibody A10G, Hölzel, Köln, Germany) diluted 1:200 in blocking solution followed by incubation with antibody anti-GADPH (GAPDH (14C10) rabbit monoclonal antibody 2118, Cell signalling), diluted 1:100 in blocking solution. Cells were then washed five times with 0.1% Tween-20 in PBS and stained for 60 min with the corresponding secondary antibodies (goat anti-mouse IgG antibody coupled to Alexa Fluor^TM^ 488 and goat anti-rabbit IgG antibody coupled to Alexa Fluor^TM^ 647, Thermo Fisher Scientific, Dreieich, Germany), diluted 1:1000 and 1:1000 in blocking solution, respectively. After several washes with 0.1% Tween-20/PBS, the coverslips were mounted on glass slides using Immunoselect Antifading Mounting Medium DAPI (Dianova^TM^, Biozol, Germany) and stored at RT until use (usually the next day). Laser scanning confocal imaging was carried out on a Leica inverted confocal microscope using a 63×/1.4 NA oil immersion objective (Leica). To visualize Alexa Fluor^TM^ 488 fluorescence, cells were excited with a 488 nm argon laser, and the emitted light was monitored between 500 and 540 nm. Alexa Fluor^TM^ 647 was excited using a 633 nm laser, and emission was detected in the 640–700 nm range. Detection of DAPI was performed using a 405 nm laser, and emission was collected from 415 to 450. For presentation, images were exported and formatted using Fiji ImageJ v2.16.0/1.54p [65].

### 5.9. Electrophysiology

Whole-cell patch clamp recordings were performed using an EPC-10 amplifier and the PatchMaster software v2x73.4 (HEKA Elektronik). Barium ions (Ba^2+^) were used as the charge carrier for the study of Ca_V_-mediated currents. The external solution (ES) contained 145 mM TEA-Cl, 10 mM BaCl_2_, 1 mM MgCl_2_, 10 mM HEPES and was adjusted to pH 7.4 with TEA-OH. The internal (pipette) solution contained 130 mM CsCl, 5 mM TEA-Cl, 10 mM HEPES, 10 mM EGTA, 2 mM Mg-ATP, 0.2 mM Na-GTP and was adjusted to pH 7.4 with CsOH. Glass pipettes with resistances of 1–2 MΩ were pulled on a Sutter P-1000 puller (Harvard Apparatus) and polished with a glowing filament using a Narishige MF-830 microforge. Data were analyzed with FitMaster v2x90.2 (HEKA) and Python version 3.8 using common libraries including NumPy v2.2.0, Pandas v2.2.3, matplotlib v3.10.0, SciPy v1.15.1, and Seaborn v0.13.2. The currents were leak subtracted, and series resistance compensation was applied. For toxin application, purified CB were diluted in the external solution, and the cells were detached from the bottom and moved to a perfusion stream containing the diluted toxin. Recordings were performed under continuous perfusion of the cell, either with ES or ES containing the venom fraction. All data are presented as the mean ± SEM.

### 5.10. Sample Preparation for Mass Spectrometry

CB containing gel bands from the captive individuals were excised, dehydrated, cut into small pieces, reduced in 20 mM dithiothreitol for 30 min at 56 °C, cooled to RT, and then alkylated with 10 mM iodoacetamide for 30 min in the dark. The Lataxan CB protein was dissolved and denatured in 6 m guanidine, buffered with 100 mM HEPES to pH 7.2, cysteine residues reduced with 10 mM DTT and alkylated with 30 mM IAA. All samples were digested with proteomics-grade trypsin, GluC and LysC at an enzyme: proteome ratio of 1:100 for 16 h at 37 °C. Peptides were desalted using home-made C18 STAGE tips [66], dried in a vacuum concentrator and reconstituted in 0.1% formic acid prior to LC/MS analysis.

### 5.11. LC/MS/MS and Data Analysis

LC–MS/MS analysis was performed with an UltiMate 3000 RSCL nano-HPLC system (Thermo Fisher Scientific, Dreieich, Germany) online coupled to an Impact II Q-TOF mass spectrometer (Bruker, Billerica, MA, USA) via a CaptiveSpray Ion Source boosted with an ACN-saturated nitrogen gas stream. An estimated 1 µg of desalted peptides was loaded on a µPAC reverse phase trap column (PharmaFluidics, Gent, Belgium ) and separated on a 50 cm µPAC reverse phase analytical column (PharmaFluidics, Gent, Belgium) operated at a column temperature of 40 °C. Peptides were eluted with a flow rate of 600 nL min^−1^ using a binary gradient from 2 to 30% eluent B (A: 0.1% formic acid in water, B: 0.1% formic acid in ACN) with 90 min effective separation time and a total runtime of 2 h per sample. Separated peptides were ionized using a CaptiveSpray nano-ESI source (Bruker) with the nitrogen gas saturated with ACN using a nanobooster (Bruker) and introduced to an Impact II high-resolution QqTOF mass spectrometer (Bruker) as described [67]. Mass spectrometry data were acquired with the Bruker HyStar Software (v5.1, Bruker) in line-mode in a mass range from 200 to 1750 *m*/*z* at an acquisition rate of 5 Hz. The 14 most intense ions were selected for fragmentation, with fragment spectra automatically acquired between 5 and 20 Hz depending on the precursor intensity. Selected precursors were excluded for the next 0.4 min unless signal to noise ratio improved 3-fold. Acquired tandem mass spectra were queried with MaxQuant v.2.4.2 [68] against a Serpentes database (release 04/2023). The fragments from the protease digestions were detected by MaxQuant v.2.4.2 using the default setting of a minimum of peptide length of seven amino acids, which provides the best balance between specificity and identification accuracy.

### 5.12. Mass Determination of Intact Protein

An Agilent HPLC-ESI-QTOF-MS system was used for mass determination of intact protein. The HPLC (Agilent 1260 Infinity series, Waldbronn Germany) system consisted of a binary pump system, an autosampler, a thermostatted column compartment and a 6250 accurate-mass QTOF-MS with an electrospray ionization (ESI) interface with a resolution of 20,000 (Agilent, Waldbronn, Germany). Chromatographic separation was performed on a ZORBAX Eclipse Plus C8 Rapid Resolution, 100 × 4.6 mm, 3.5 µm (Agilent, Waldbronn, Germany). Column temperature was kept at 50 °C. Flow rate was 700 μL/min. The mobile phase consisted of solvent A, which was 0 1% FA in water, and solvent B, which was 1% FA in ACN. Sample injection volume was 20 μL. At the beginning of the run was an isocratic step of 5% B (0–5 min) followed by an increase to 60% B within 20 min. Afterwards, the gradient increased rapidly within 0.5 min to 95% B and was held for cleaning with 95% B for 4.5 min. The gradient returned to 5% B within 0.1 min and equilibrated the system for 4 min. Detection was performed with the QTOF mass detector in the ESI positive ionization mode. The nebulizer pressure was set to 20 psi and the drying gas flow was set to 11 L/min. A fragmentation voltage of 215 V, a skimmer voltage of 68 V and an octopole voltage of 750 V were used. The mass range was set to *m*/*z* 500–3200 and the data acquisition rate was two spectra. Source temperature was set to 300 °C. MassHunter software LC–MS Data Acquisition vB.05.01 (Agilent Technologies, Santa Clara, CA, USA) was used to control the instrument and data acquisition.

## Data Availability

The data presented in this study are openly available in a publicly accessible repository at https://jugit.fz-juelich.de/HidalgoPatricia/crotoxin_cb_cav, accessed on 15 October 2025. Raw mass spectrometry and search data have been deposited to the ProteomeXchange Consortium via the PRIDE partner repository with the dataset identifier PXD070916 [69,70].
